# Disability, Depression and Disc Surgery: Lets be Careful with the Evidence

**DOI:** 10.5812/kowsar.22287523.2015

**Published:** 2011-09-26

**Authors:** Muhammad Shahzad Shamim

**Affiliations:** 1 Neurosurgery Department, Aga Khan University Hospital, Karachi, Pakistan

**Keywords:** Depression, Low-back pain, Discectomy

*Dear Editor*,

I read with interest the article by Farzanegan et al. , titled “Effects of lumbar discectomy on disability and depression in patients with chronic low back pain” (1). It was a very well-written article on an important subject, which has remained largely ignored. I would therefore like to congratulate the investigators for highlighting the subject. However, I have a few concerns regarding the methodology of the study. First, I am not convinced that discectomy or laminectomy is ever indicated for back pain alone. It is also unclear whether these operations can lessen chronic back pain; they could also aggravate the pain. The best surgical outcomes after discectomy or laminectomy are expected in cases of radicular leg pain that is unresponsive to the best medical management available (2). While there are cases where patients have reported alleviations in their symptoms of back pain, this cannot be interpreted as the goal of the surgical intervention. Second, the study attempts to establish a cause–effect relationship between lumbar discectomy and disability and depression in patients with chronic low back pain. Even though lumbar discectomy has been shown to provide short-term reduction in the disability, several studies have shown that the long-term results did not largely differ among patients who chose to undergo surgery for their symptoms of back pain or those who chose the non-operative treatment option. Therefore, to establish a definite cause–effect relationship between a surgical procedure and disability or depression, similar studies should be performed with control subjects. Notably, a high frequency of depression was observed in patients (70%) who underwent this surgical procedure than in patients who underwent other similar surgical procedures (3, 4). Finally, the study does not mention the type of perioperative care offered to the patients, whether similar care was provided for all patients, or whether patients with differences in perioperative care, including earlier mobilization, physiotherapy, or administration of anti-depressants, showed improvement in symptoms. These perioperative care options should be considered because approximately 40% patients who experience symptoms of chronic back pain are likely to present with moderate to severe depression that requires addressing by means other than discectomy or laminectomy. Hence, although the study is interesting, the methodology used in the study was too simplistic and was not sufficient to answer some important questions.

## References

[1] Farzanegan G, Alghasi M, Safari S, Ahmadi SA (2011). Effects of lumbar discectomy on disability and depression in patients with chronic low back pain.. Anesth Pain..

[2] Shamim MS, Parekh MA, Bari ME, Enam SA, Khursheed F (2010). Microdiscectomy for lumbosacral disc herniation and frequency of failed disc surgery.. World Neurosurg..

[3] Lebow R, Parker SL, Adogwa O, Reig A, Cheng J, Bydon A (2011). Microdiscectomy improves pain-associated depression, somatic anxiety, and mental well being in patients with herniated lumbar disc.. Neurosurgery..

[4] Shamim MS, Enam SA, Qidwai U (2009). Fuzzy Logic in neurosurgery: predicting poor outcomes after lumbar disk surgery in 501 consecutive patients.. Surg Neurol..

